# Remarks on Mr. Hope’s Camphor Mixture in Dysentery

**Published:** 1838-08-29

**Authors:** C. D. Meigs

**Affiliations:** Lecturer on Midwifery, &c.


*Remarks on* Mr. Hope's *Camphor Mixture in Dysentery.* By C. D. Meigs, M. D., Lecturer on Midwifery, &c.

To the Editors of the Medical Examiner.

*Gentlemen:*—I have seen in a late number of your valuable journal, some observations on dysentery in a lecture by Dr. Gerhard. In that lecture he adverts to the use of Hope's Camphor Mixture, and dwells on it with less emphasis than appears to me desirable. Hence I beg leave to trouble you with the following remarks.

Thomas Hope, Esq., a British Naval Surgeon, published in the Ed. Med. and Surg. Journal for July, 1826, "Observations on the powerful effects of a Mixture containing Nitrous Acid and Opium in curing Dysentery, Cholera, and Diarrhœa." His paper bears date "Chatham, April 19th, 1826." He says, "In March, 1800, I communicated in a letter to John Pearson, Esq., of Golden Square, the discovery of this useful remedy, and my observations were printed in the 3d vol. of the Lond. Med. Journal, and afterwards in the 2d vol. of the Edinburgh Practice of Physic, published in the year 1803."

Dr. H. states that since the above period he has continued the use of the remedy with unvaried success, and among other evidences of its power he relates that, in 1815, a medical officer of rank put under his care five men who were invalided in hospital, who were deemed incurable, had taken the sacrament, and were quietly waiting their fate. The medicine was administered and cured three of them. In 1819, twenty-six men were invalided for dysentery, from the West Indies, and divided into wards, eleven in one and fifteen in the other. It was agreed that the fifteen should be treated with the acid mixture, and the eleven in the usual manner. Of the fifteen patients, twelve were cured, and three died:—whereas, of the eleven patients, three recovered and eight died.

Mr. Hope gives many other very encouraging relations of his success, which it is not, perhaps, necessary to cite here, but which render his paper so interesting that I should be very much pleased if it could be republished entire in this country. I will, however, cite the fact that on board the Ganymede frigate, in 1821, many cases of cholera and diarrhœa were successfully treated. Also that on board the Dolphin, in 1824, he treated seventy-one cases of disorder of the bowels, many of them remarkably severe. Not one of the patients died. Not one of them had occasion for more than five doses, and many required only two. On board the same ship, in July, 1825, two hundred and sixty-four cases of cholic, dysentery, cholera, and diarrhœa occurred. Not one of these died, which he attributes as striking evidence of the curative effects of the acid mixture.

The formula for the composition of the medicine as used in all Mr. Hope's cases is as follows:—

℞. Acidi Nitrosi, f℈i.

Mist. Camphoræ, f℥viii.

Misce et adde

Tinct. Opii, gtt. xi.

Sig. One fourth part to be taken every three or four hours.

He says, "A small addition of syrup of red poppies improves not only the appearance of the mixture, but, in some instances, it has appeared to increase its effects."

Mr. Hope considers that no previous preparation is required for its exhibition in chronic dysentery. The dose of two ounces three times a day is quite sufficient; in these cases he recommends that the hands and feet should be kept warm while using the medicine, and that the body should be preserved as much as possible from exposure to extreme cold, or currents of air. The patient should make use of warm barley water or thin gruel, and a diet of sago and tapioca.

Mr. H. *insists* on the use of *nitrous* and not *nitric* acid in the formula.

Such, Messrs. Editors, is the too short abstract which I have made of Mr. Hope's valuable paper. I wish now to say, that I was attending a carpenter named Miller, in this city, I think in September, 1826. I had exhausted all my means of cure in his case,—viz. venesection, calomel, and opium, emulsions of oil, anodyne enemeta, &c., and, after many days of intense suffering, he was still tormented with tormina and tenesmus which called him up from thirty to forty times per diem. I was exceedingly doubtful as to his safety, when I received my July number of the Edinb. Med. and Surg. Journal containing Mr. Hope's paper. I read it, procured the acid mixture, he took eight doses and was well thenceforth. Since that time I have habitually resorted to the use of Mr. Hope's medicine. I have recommended it to my medical brethren, and it has come to be much in use in our city. But it is given in doses too small, and hence the rather faint praise which it meets at the hands of some of our medical gentlemen. I am ready on my part to avow that I have less reason to feel disappointment, after adopting Mr. Hope's practice, than I usually have had, upon trying methods boldly vaunted by the writers in our medical journals. I have used it with the most happy results in numerous cases of cholera, of ordinary cholera morbus, diarrhœa, dysentery, and cholera infantum. I think it fully deserving of all the commendations lavished on it by Mr. Hope, and I earnestly desire that the readers of your useful work may make a fair trial of it in the dysenteries which now prevail.

I shall add, that regarding mœlina as a discharge from the liver, and, therefore, possessing some remote resemblance to cholera morbus, I have in several very dangerous cases of that disease, attended with profuse hematemesis, prescribed the Hope mixture, and always with the happiest result.

Very respectfully your friend, and servant,

C. D. Meigs.

